# Impact of CuSn10 Powder on Mechanical Properties and Tribological Performance of Novel Basalt Fiber-Reinforced Hybrid Composites

**DOI:** 10.3390/polym17091161

**Published:** 2025-04-24

**Authors:** Corina Birleanu, Bere Paul, Razvan Udroiu, Mircea Cioaza, Marius Pustan

**Affiliations:** 1 MicroNano Systems Laboratory, Mechanical Systems Engineering Department, Technical University from Cluj-Napoca, Blv. Muncii nr. 103-105, 400641 Cluj-Napoca, Romania; corina.barleanu@omt.utcluj.ro (C.B.); mircea.cioaza@staff.utcluj.ro (M.C.); marius.pustan@omt.utcluj.ro (M.P.); 2 Manufacturing Engineering Department, Technical University from Cluj-Napoca, 400641 Cluj-Napoca, Romania; 3 Manufacturing Engineering Department, Transilvania University of Brasov, Blv. Eroilor nr. 29, 500036 Brașov, Romania; udroiu.r@unitbv.ro

**Keywords:** tribology, hybrid composite, basalt fiber, CuSn10, mechanical properties, abrasive wear, statistical analysis, GLM method

## Abstract

Hybrid composite materials reinforced with both fibers and particulate fillers are increasingly used in engineering due to their favorable balance of mechanical strength, reduced weight, and enhanced tribological performance. This study investigated the effect of CuSn10 bronze powder additions (5%, 10%, and 15% by weight) on the mechanical and tribological properties of novel basalt fiber-reinforced polymer (BFRP) composites. The composites were fabricated via vacuum-assisted processing and tested under dry sliding conditions with varying loads (10, 20, and 30 N) and sliding speeds (0.1, 0.25, and 0.36 m/s). The results show that the optimal tensile strength (440.6 MPa) was achieved at 10 wt% CuSn10, while the best tribological performance was observed at 15 wt% CuSn10, under a 10 N load and 0.25 m/s sliding speed, where the coefficient of friction decreased by up to 38% and the specific wear rate was reduced by more than 50% compared to the unreinforced BFRP composite. These enhancements are attributed to the formation of a stable oxide-based tribolayer (CuO, SnO_2_) and improved load transfer at the fiber–matrix interface. Statistical analysis (GLM) confirmed that CuSn10 content had the most significant influence on tribological parameters. The findings provide valuable insight into the design of high-performance hybrid composites for structural and tribological applications.

## 1. Introduction

The performance of these materials is directly influenced by their internal structure, the nature of the reinforcement, and the spatial distribution of the reinforcement within the polymer matrix. Traditional materials such as metals and ceramics are gradually being replaced by composite materials in industries like aerospace, automotive, and marine due to their enhanced strength-to-weight ratio and reduced production costs. Polymer composites reinforced with fibers (FRPs) have shown remarkable resilience when subjected to dynamic stresses, elevated temperatures, and chemically harsh conditions, which makes them ideal candidates for use in challenging industrial environments. Moreover, the ability to tailor composite properties by adjusting the fiber type, matrix composition, and manufacturing process offers significant advantages in product design and performance optimization.

Basalt fiber-reinforced polymer (BFRP) composites have emerged as a promising alternative to glass fiber-reinforced polymer composites (GFRP) composites due to their higher thermal stability, greater chemical resistance, and improved environmental friendliness. Basalt fibers are produced from natural volcanic rock, making them more cost-effective and sustainable compared to synthetic fibers. BFRP composites have demonstrated excellent tensile strength, impact resistance, and fatigue performance, making them ideal for structural and tribological applications. However, despite these advantages, BFRP composites still face limitations related to friction and wear performance under high-load and high-speed sliding conditions. The relatively poor self-lubricating properties and susceptibility to adhesive wear under certain operating environments restrict their broader application in tribological systems.

In a study by Salim et al. [1], three distinct fiber reinforcements were integrated into an epoxy matrix to fabricate composite laminates: basalt-woven fibers and AISI304 stainless steel wire meshes with densities of 100 and 200, respectively. The mechanical performance of these hybrid laminates was evaluated through statistical analysis, which identified the optimal configuration in terms of flexural strength and modulus. The study reported an increase in flexural strength and modulus of up to 25% compared to plain composites.

Rong et al. [2] utilized an electrostatic self-assembly technique to deposit graphene oxide (GO) onto basalt fiber (BF) fabric surfaces, aiming to improve the interfacial adhesion in basalt fiber-reinforced polyamide 6 (PA6) composite laminates (BF/PA6). Their results demonstrated that the electrostatic assembly of GO effectively enhanced the surface roughness, wettability, surface energy, and chemical reactivity of the basalt fiber fabric, all the while maintaining the structural integrity of the fibers. At a GO concentration of 0.8 g/L, the BF/PA6 composites exhibited increases of 85.1% in interlaminar shear strength, 43.2% in impact strength, 53.2% in flexural strength, and 63.4% in flexural modulus. Additionally, the average friction coefficient and wear rate decreased by 15.2% and 40.5%, respectively. The research [3] introduced a new type of epoxy composite (EP/BF/RGO/PW) that incorporates basalt fiber, reduced graphene oxide, and paraffin wax. This hybrid reinforcement approach represents an innovative strategy designed to boost both the mechanical strength and tribological performance of epoxy-based composites. The findings indicate that incorporating basalt fibers leads to notable improvements in these properties.

In [4,5,6,7,8], a thorough review of recent tribological tests on fiber-reinforced composites (FRCs) was provided, focusing on their performance under varying load and velocity conditions. This comprehensive analysis helps in understanding the complex interactions that affect wear and friction of these materials. The study [9] indicated that basalt fiber reinforcement significantly enhances wear resistance and thermal stability in friction composites. The addition of hard fillers like silicon carbide further improves mechanical properties, reducing specific wear rates and enhancing friction performance under sliding conditions. Modified basalt-reinforced epoxy composites [10] utilize functionalized, carboxylated, multi-walled carbon nanotubes and polydopamine to enhance interfacial properties, resulting in significant improvements in tensile, bending, and impact performance, making them suitable for aerospace, automotive, and construction applications. The application of 3-aminopropyltriethoxysilane (APTES) reduces the surface energy of basalt fibers, enhancing their dispersion and interfacial strength with epoxy, leading to increased tensile and flexural strengths by 55.87% and 36.36%, respectively (Xiang et al.) [11].

To address the limitations of BFRP composites in friction and wear applications [12,13,14], metallic fillers have been widely explored as reinforcement additives. Copper (Cu) has shown promise as a filler due to its high thermal conductivity, excellent load-bearing capacity, and ability to enhance interfacial bonding with the polymer matrix. Previous research [15] has demonstrated that incorporating Cu powder into fiber-reinforced composites improves tensile strength, wear resistance, and friction stability under sliding conditions. The presence of Cu enhances the load transfer capacity at the fiber–matrix interface, reduces surface wear, and minimizes the COF due to its high ductility and thermal stability. In a previous study conducted by the authors [15], the addition of Cu powder to basalt fiber-reinforced epoxy composites led to significant improvements in both mechanical and tribological performance. However, certain challenges related to oxidation and long-term friction stability under varying environmental conditions remain unresolved.

To further improve the tribological performance of BFRP composites, this study explored the use of bronze (CuSn10) powder as an alternative to pure copper. CuSn10 is a bronze alloy consisting of 90% copper and 10% tin, offering distinct advantages over pure copper. Incorporating tin increases the material’s hardness, improves its wear resistance, and imparts self-lubricating characteristics by promoting the formation of a thin oxide layer during sliding contact. Bronze alloys are also known for their excellent corrosion resistance, making them suitable for harsh operating environments. The self-lubricating effect of tin reduces adhesive wear and lowers the coefficient of friction, thereby improving the sliding contact performance of the composite material. Therefore, CuSn10 represents a strategic choice for improving the mechanical strength and tribological behavior of basalt fiber-reinforced hybrid composites.

While the literature provides numerous studies on optimizing polymer composites using fibers or metallic fillers, most of them examine either mechanical or tribological properties in isolation. Moreover, there is a noticeable lack of research focusing specifically on the effects of CuSn10 bronze powder reinforcement in basalt fiber-reinforced composites, especially under dry sliding conditions with variable loads and speeds.

Using a full factorial experimental design and advanced statistical methods (GLM), this study simultaneously evaluates tensile, flexural, and frictional performance under different sliding speeds and applied loads. The results provide valuable insights into the development of high-performance hybrid composites suitable for industrial applications where durability and frictional stability are critical.

The main goal of this research was to examine how the incorporation of CuSn10 influences the mechanical and tribological behavior of hybrid composites reinforced with basalt fibers. The research will evaluate key mechanical properties, including tensile and flexural strength, as well as tribological parameters such as the COF, wear rate, and contact temperature under varying load and sliding speed conditions [16,17,18].

The specialized literature indicated multiple studies exploring methods for optimizing composite materials [19,20,21,22,23,24], especially fiber-based ones, through the incorporation of micro- and nanoparticles. However, some sources emphasized the limited number of studies regarding the use of powders as modifiers for polymer composites [25,26,27,28,29]. In this context, the present study aimed to contribute to expanding knowledge on the effects of metallic powders on the mechanical and tribological performance of basalt–epoxy composites.

This study tested the hypothesis that the addition of CuSn10 will improve the mechanical properties of BFRP composites by enhancing interfacial bonding and load transfer capacity. Additionally, it is expected that the presence of tin in CuSn10 will reduce adhesive wear and improve the self-lubricating properties of the composite during sliding contact. Furthermore, CuSn10 reinforcement is anticipated to result in lower COF and improved wear resistance under high-load and high-speed sliding conditions compared to Cu-reinforced composites.

This research aimed to provide valuable insights into the design and development of high-performance basalt fiber-reinforced composites for structural and tribological applications. The findings are expected to advance composite materials with enhanced mechanical strength, friction stability, and wear resistance.

By comparing the results with previous findings on Cu-based composites, this study sought to clarify the role of CuSn10 in improving the mechanical and tribological behavior of basalt fiber-reinforced hybrid composites. This research investigated a novel approach that combines vacuum bag molding with the controlled integration of CuSn10 powder at various weight percentages, an area insufficiently addressed in current studies. Additionally, the application of statistical analysis and 3D microscopy in investigating wear mechanisms provides new insights into the formation of protective copper-based tribofilms during sliding contact. These insights are anticipated to advance the understanding of hybrid composite behavior and support the development of high-performance materials for aerospace, automotive, and broader industrial applications.

## 2. Materials and Methods

### 2.1. Materials

The composite materials used in this study were prepared using a carefully selected combination of basalt fiber fabric as reinforcement, epoxy resin system, and CuSn10 bronze powder as metallic filler.

**Figure 1:**
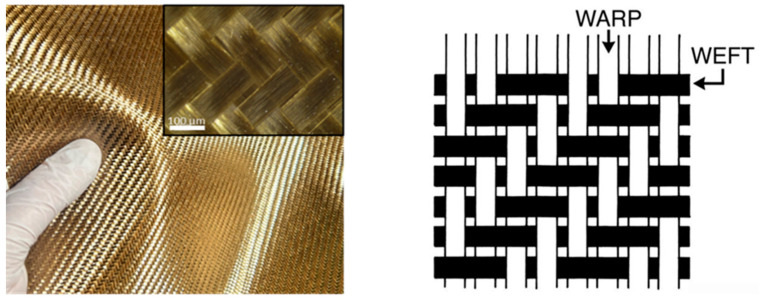
Twill 2/2 BF weaves used in the fabrication of the studied composite materials.

The reinforcement used in the composite fabrication was basalt fiber fabric with a surface density of 220 g/m^2^ in a twill weave configuration (Figure 1). The epoxy matrix employed in this study consisted of EPIKOTE™ Resin MGS™ LR 135 resin (HEXION GmbH, Duisburg, Germany) mixed with the corresponding EPIKURE^TM^ MGS^TM^ Curing Agent LH136 (HEXION GmbH, Duisburg, Germany) at a precise weight ratio of 100:35. As a metallic filler, CuSn10 powder (Easy Composites Ltd., Stoke-on-Trent, UK) with a mean particle size of 74.5 µm was incorporated into the composite formulation. According to the supplier’s technical data sheet, the bronze powder has an irregular, non-spherical morphology typical of gas-atomized or mechanically processed bronze alloys. Specific surface area values were not provided. The chemical composition of the powder includes between 10.0% and 12.0% tin, with the remainder being copper.

Composite samples consisted of five layers of basalt fiber fabric, measuring 500 mm × 300 mm and having a weight fiber fraction (wf) of 50%, CuSn10 powder at concentrations of 5%, 10%, and 15%, and epoxy resin. Table 1 presents the composition of the laminated composites samples.

A reinforcement degree of 50% represents an optimal compromise between cost and mechanical properties. Although glass and basalt fibers are relatively expensive, this level of reinforcement provides a significant improvement in mechanical strength compared to unreinforced materials while keeping costs at a sustainable level. As an environmentally friendly material, basalt fabric is made from a natural resource, has a low environmental impact and does not emit toxic substances during use. Due to its excellent compatibility with epoxy matrices, this material ensures superior adhesion and uniform distribution of mechanical stresses in the final composite.

For the tribological tests, bearing balls made of high-quality chrome steel 52100 (RKB Bearing Industries Group, Balerna, Switzerland) were used as counterparts. The balls are similar to those used in [14,15,16,17].

### 2.2. Methods

#### 2.2.1. Fabrication of Sample Composites

The fabrication of the basalt fiber-reinforced composites with CuSn10 bronze powder involved a controlled process to ensure consistent quality and reliable mechanical performance. A combination of hand lay-up impregnation and vacuum bagging was used to achieve uniform fiber wetting and remove air bubbles, ensuring a void-free composite structure.

First, the epoxy resin system (EPIKOTE™ MGS LR 135 and EPIKURE™ MGS LH136) was thoroughly mixed at a 100:35 weight ratio. The CuSn10 bronze powder was gradually added to the resin mixture at weight fractions of 5%, 10%, and 15%, ensuring even dispersion using mechanical stirring. Five layers of basalt fiber fabric (220 g/m^2^, twill weave) were carefully laid in a metal mold (500 × 300 mm). The resin mixture was applied between each layer using a hand lay-up method to ensure complete impregnation of the fibers.

**Figure 2:**
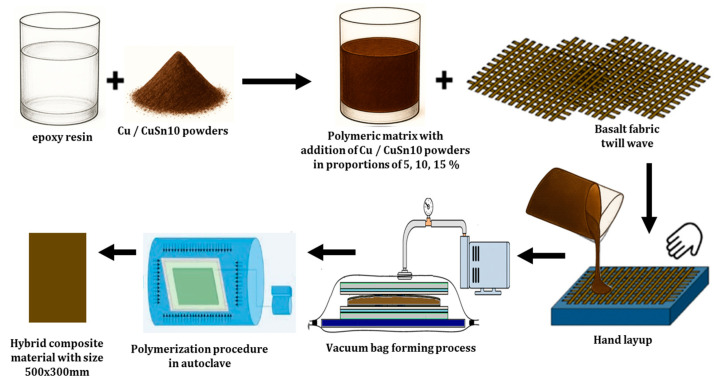
The manufacturing process of hybrid composite materials.

After lay-up, the composite stack was enclosed in a vacuum bag and sealed under controlled pressure. The vacuum bagging process removed trapped air and excess resin, promoting consistent fiber distribution and minimizing void formation. The assembled lay-up was subsequently placed in a Maroso autoclave (Maroso SRL, Pianezze VI, Italy) and subjected to a controlled curing cycle, consisting of 180 min at 120 °C under a vacuum pressure of −0.9 bar and an internal pressure of 4 bar. The cured composite was slowly cooled to 30 °C over a period of 60 min to prevent thermal stress and ensure dimensional stability, as depicted in Figure 2.

**Figure 3:**
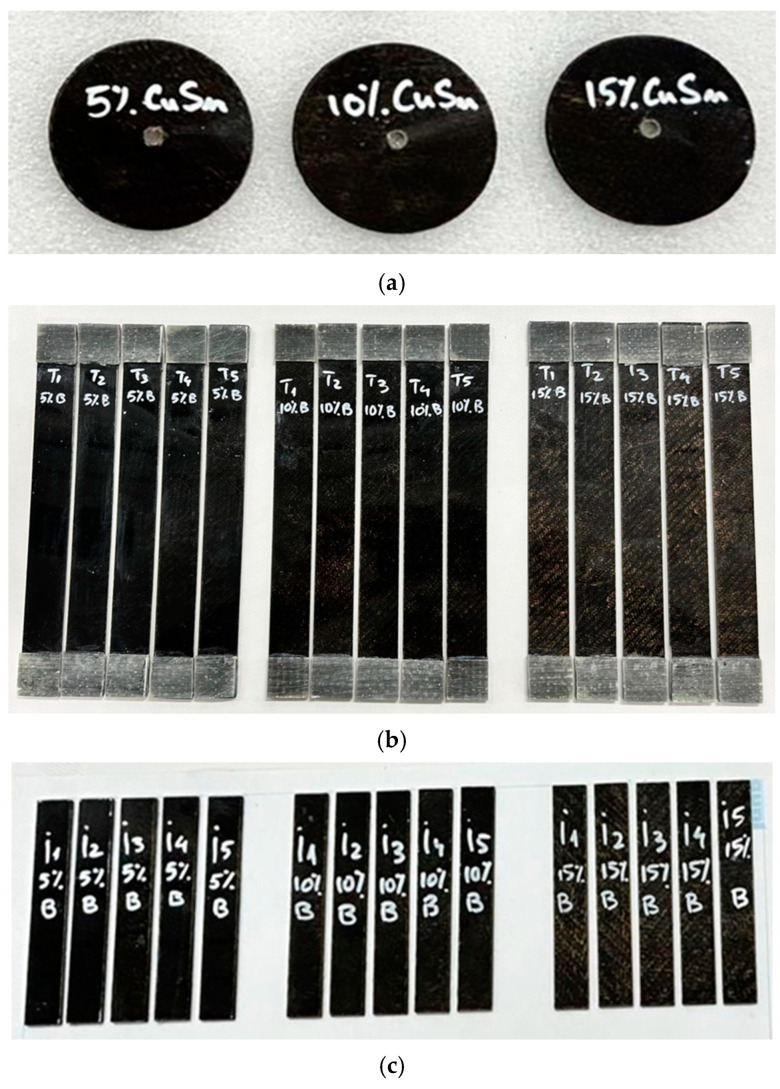
Specimens made of polymer composite materials reinforced with basalt fibers and metallic powders (CuSn10) used in (**a**) tribological experimental testing; (**b**) tensile mechanical testing; (**c**) flexural mechanical testing.

Following the cutting and finishing process, standardized samples necessary for testing the properties of the composites were obtained:

- Disks for tribological tests with a diameter of 50 mm and thickness of 2 mm, intended for wear resistance analysis (Figure 3a);
- Samples for tensile tests (according to ASTM D3039-17 [24]) measuring 250 mm × 25 mm × 2 mm, used to determine tensile strength (Figure 3b);
- Samples for flexural tests (as per ASTM D7264D [30]), measuring 80 mm × 13 mm × 2 mm, aimed at evaluating bending behavior.

#### 2.2.2. Mechanical Tests of Basalt Fiber-Reinforced Hybrid Composites

The specimens used for tensile tests had a rectangular cross-section including a calibrated zone and specially designed ends for gripping in the testing device. Mechanical testing was performed using an INSTRON 8801 universal testing machine (Instron, Norwood, MA, USA). The specimens were loaded at a crosshead speed ranging from 2 to 4 mm/min and subjected to tensile stress until failure occurred. Flexural tests were conducted on an Instron 3366 universal testing machine (Instron, Norwood, MA, USA), with a maximum load capacity of 10 kN.

These trials were conducted using the three-point bending method, with testing parameters similar to those used in tensile tests.

Five specimens were used for each test and the repeatability of the tests was determined based on the coefficient of variation (CV). The coefficient of variation (CV) was determined using the standard deviation and mean values, as described in reference [18]. All experiments were carried out under controlled room temperature (20 °C) and at 50% relative humidity. Based on these experimental tests, essential mechanical properties were determined, including specific elongation, fiber expansion at fracture, yield strength, ultimate tensile strength, and modulus of elasticity. Analyzing these parameters allows for evaluating the quality of the manufacturing process, as variations in material composition or production technology may have a substantial effect on the mechanical behavior of the composite.

#### 2.2.3. Tribological Tests of Basalt Fiber-Reinforced Hybrid Composites

**Figure 4:**
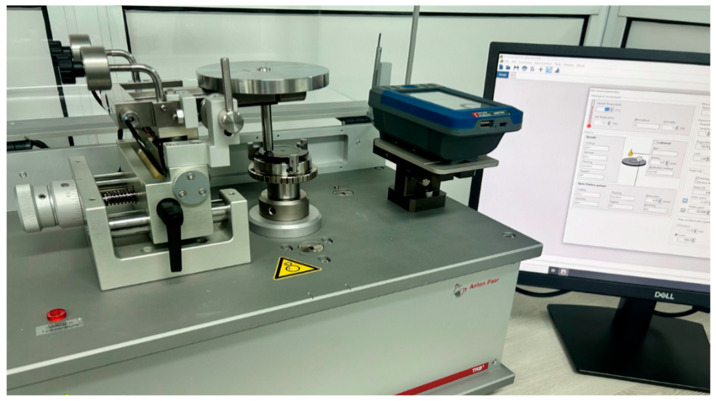
TRB^3^ pin-on-disk tribometer.

Tribological research was conducted using TRB3 tribometer (Anton Paar GmbH, Graz, Austria) depicted in Figure 4. TRB^3^ complies with ASTM G99 [31], ASTM G133 [32], and DIN 50324 [33] standards, ensuring that tribological tests are conducted according to best industrial practices. Regular calibration of the tribometer was performed to maintain the accuracy and reliability of the measurement data. The software of the tribometer allows for setting parameters to meet all testing needs (different modes of speed, loading, movement, etc.). Additionally, the software includes a “Modelization” module by default, which allows for simulation of contact stress and deformation distribution.

The tribological experiment was carried out following a similar protocol as in [22]. The use of AISI 52100 steel as a counterface material is well established in tribological studies due to its high hardness, dimensional stability, and wear resistance, making it a reliable reference material. Compared to softer counterfaces (e.g., aluminum alloys) or ceramic materials (e.g., Al_2_O_3_), the AISI 52100 pairing better simulates conditions in bearings, shafts, or wear-prone interfaces in mechanical systems, allowing for consistent evaluation of material wear behavior under realistic contact conditions. In the tribological analyses, the focus was on characterizing abrasive wear, material transfer, and the thermal stability of the friction pairs. Accurate definition and control of the operating parameters, as outlined in Table 2, are crucial to ensuring the reliability and repeatability of the experimental results. The friction coefficient was recorded and the wear rate was quantified, providing relevant data on the tribological performance of the studied materials. For comprehensive analysis of the underlying wear mechanisms, microscopic images of the tested surfaces were taken, experimental parameters were recorded, and the material transfer phenomenon was analyzed.

Tribological parameters, such as friction coefficient and wear rate, are strongly influenced by testing conditions, and variations under these conditions can significantly affect the interpretation of experimental results. Essential parameters, such as humidity and room temperature, as well as the local temperature in the contact area, were continuously monitored.

Test validation was achieved by ensuring experimental repeatability, with five trials conducted for each set of conditions. This was complemented by continuous monitoring of environmental parameters, regular equipment calibration, and calculation of the coefficient of variation (CV) to assess data consistency.

Precise assessment of the wear track is critical for reliable functional evaluation. Although profilometric techniques are particularly effective for such measurements, they can be prone to errors. Variables such as the operator’s interpretation and the applied measurement methodology can considerably affect the accuracy and consistency of the wear track analysis.

The most effective approach consists of conducting a comprehensive assessment of the wear track surface. In pin-on-disk testing configurations, the number of profilometric scans is a key factor in accurately characterizing the wear track, with a greater number of scans required in cases where the wear pattern is non-uniform [19,20,21,22,23].

**Figure 5:**
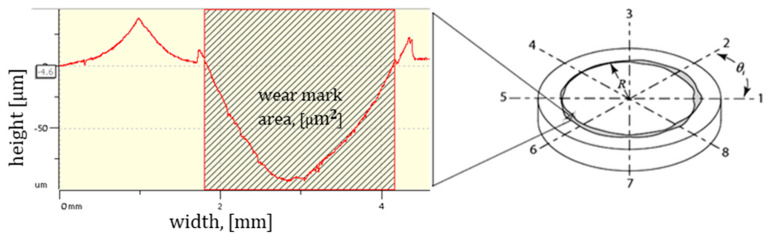
Schematic diagram of the wear track formed during the pin-on-disk experiment.

Each disk was subjected to eight profilometric scans, spaced at 45° intervals (Figure 5), enabling a more accurate estimation of volumetric wear and minimizing measurement uncertainty. Although a minimum of four scans is typically recommended to optimize measurement time in practical applications [25], the increased number of scans in this study enhanced reliability. The findings of this study showed that the weight loss of the chromium-alloyed steel balls was minimal and could be considered negligible, so the effects of ball wear were interpreted only through SEM analyses of the wear tracks carried out in each subsection of this work.

The tribological parameters—coefficient of friction (COF) and specific wear rate (K)—were evaluated according to the procedure outlined in references [14,17]. Additionally, the sample temperature (T) during testing was monitored using a FLIR E5xt infrared thermal camera (Teledyne FLIR Company, Wilsonville, OR, USA), which features MSX and Wi-Fi capabilities. In this study, an experimental design involving three control factors—weight fraction (wf), applied force (F), and sliding speed (v)—was implemented to examine their effects on three response variables: coefficient of friction (COF), specific wear rate (K), and temperature (T). The control factors and their corresponding levels are listed in Table 3. A full factorial experimental design with 36 combinations was implemented to assess the effects of the control factors on the response variables. Statistical analysis was performed using Generalized Linear Models (GLMs) and multifactorial ANOVA, utilizing Minitab 19 software (Coventry, UK). The significance of each control factor and its percentage contribution (PC%) were determined from the ANOVA tables, based on Fisher’s F-value, *p*-value, and the sequential sum of squares. Graphical methods—including main effects plots, interaction plots, and interval plots of the response variables versus all control factors—were used to visualize the data, and the assumptions of ANOVA were validated accordingly.

#### 2.2.4. Morphology Analysis of Basalt Fiber-Reinforced Hybrid Composites

The JEOL JSM-5600LV Scanning Electron Microscope (JEOL Ltd., Tokyo, Japan) was used to analyze the wear track and the subsequent determination of the wear rate or wear volume. Elemental analysis was conducted using an ULTIM MAX 65 energy-dispersive X-ray spectroscopy (EDX) detector (Oxford Instruments, High Wycombe, UK). The analysis was conducted using Aztec 4.2 software, which facilitated precise spectral acquisition, real-time elemental identification, and high-sensitivity elemental mapping.

## 3. Results and Discussion

### 3.1. Mechanical Characterization of Basalt Fiber-Reinforced Hybrid Composites

Basalt fiber-reinforced composites with bronze powder (BFRP_CuSn10) were investigated to evaluate the influence of bronze on mechanical performance, particularly in terms of tensile behavior. The materials were manufactured with a fixed content of 50% basalt fibers, and the bronze powder was added in different proportions of 5%, 10%, and 15% of the total weight. The test results show that the addition of bronze has a positive effect on tensile strength, with a peak reached at 10% CuSn10.

**Figure 6:**
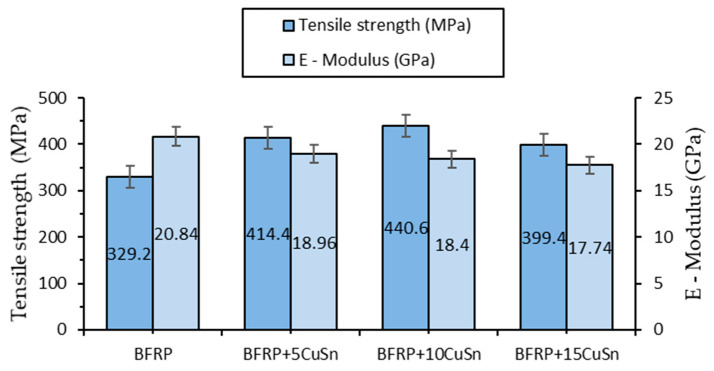
Variation of tensile strength and elastic modulus for BFRP_CuSn10 samples.

The tensile test results for each composition showed the following characteristics, presented in Table 4 and the graph in Figure 6.

Tensile strength increased from 329.2 MPa for the material without bronze to 414.4 MPa for 5% CuSn10 and 440.6 MPa for 10% CuSn10, indicating a significant improvement in the composite’s ability to withstand axial loads. However, for a concentration of 15% CuSn10, the tensile strength decreased to 399.4 MPa, suggesting that an excess of metallic particles can negatively affect the material’s structure. This decrease may be caused by potential homogeneity issues and the formation of regions with an uneven distribution of bronze in the matrix.

The specific tensile strain followed a similar trend, increasing from 1.84% for the initial material to 2.62% for 10% CuSn10, which suggests improved ductility. At 15% CuSn10, this value slightly decreased to 2.47%, which could be attributed to local stiffening caused by the excessive concentration of metallic particles. This indicates that the optimal bronze addition for simultaneously improving strength and ductility is around 10%.

The elastic modulus decreased slightly with the addition of bronze, from 20.84 GPa for the material without bronze to 17.74 GPa for 15% CuSn10, suggesting a reduction in material stiffness. The presence of bronze particles forms stress-relieving regions, contributing to increased flexibility in the material.

This decrease in stiffness may also be partially attributed to particle agglomeration at higher CuSn10 contents, which can create microstructural heterogeneities and local stress concentrations that affect load distribution. Additionally, the presence of metallic particles within the epoxy matrix may slightly disrupt matrix continuity, reducing its effective stiffness and potentially contributing to the observed drop in the elastic modulus.

In conclusion, the addition of 10% CuSn10 appears to be optimal, offering a balance between high mechanical strength, good deformation capacity, and improved homogeneity.

**Figure 7:**
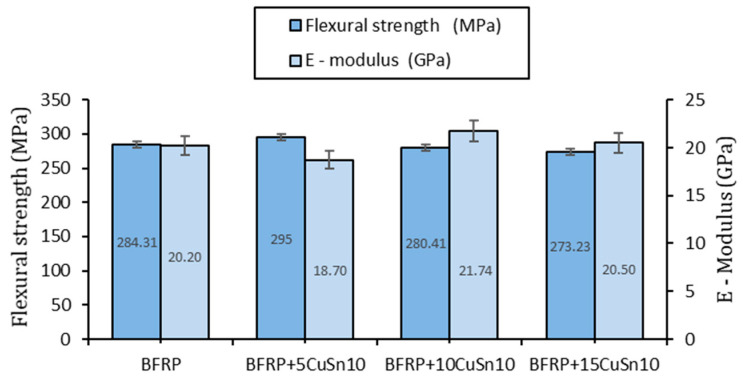
Variation of flexural strength and elastic modulus for BFRP_CuSn10 sample.

The three-point bending tests revealed the following properties, presented in Table 5 and the graph in Figure 7.

Regarding the flexural behavior, the results indicate a different trend compared to tensile behavior, with smaller variations in mechanical strength and a more complex influence of bronze addition. The material without bronze showed a flexural strength of 284.31 MPa, while the addition of bronze resulted in a slight increase to 295 MPa for 5% CuSn10, followed by a decrease to 280.41 MPa for 10% CuSn10 and 273.23 MPa for 15% CuSn10. This suggests that, unlike tensile behavior, the bronze particles do not have the same reinforcing effect under bending loads, and a higher concentration may even reduce the material’s ability to withstand such stresses.

The specific flexural strain remained relatively constant, with values between 2.22% and 2.31% for the 5% and 15% CuSn10 variants, but it dropped to 1.41% for 10% CuSn10, indicating temporary stiffening of the material at this percentage. This decrease could be associated with a different distribution of bronze within the matrix, influencing how the composite responds to perpendicular stresses.

The elastic modulus showed greater variations than in the case of tensile strength, ranging from 20.2 GPa for the material without bronze to 21.74 GPa for 10% CuSn10, suggesting that at this percentage, bronze may contribute to better stiffening of the material under bending conditions. At higher concentrations, the elastic modulus decreased to 20.5 GPa for 15% CuSn10, confirming that an excess of bronze can negatively affect the structural homogeneity of the composite.

The mechanical behavior analysis of the BFRP_CuSn10 composites shows that adding bronze significantly improves tensile strength, with a peak at 10% CuSn10, but has a less clear effect on bending strength, where changes are smaller and more variable. Although a slight increase in flexural strength was recorded at 5% CuSn10, higher concentrations led to a decrease, suggesting an optimal additive level of around 10% for maintaining a balance between mechanical properties.

The general trend indicates that the elastic modulus decreases slightly under tensile load, suggesting a reduction in stiffness and an increase in deformability but remains relatively constant under bending, which points to possible structural consolidation under perpendicular stresses. The variability of the results is low for the 10% CuSn10 variants, confirming that this is the optimal percentage for improving the homogeneity and structural stability of the composite.

In conclusion, BFRP_CuSn10 composites offer a promising combination of mechanical strength and flexibility, and tribological tests will be essential to determine the effects of bronze on wear and friction behavior. This analysis suggests that BFRP_CuSn10 could be an optimal solution for applications requiring both improved mechanical properties and good performance under dynamic loads.

Regarding the impact on tribological behavior, BFRP-CuSn stands out for its superior wear resistance due to the properties of bronze, which reduce adhesion and minimize material loss under friction conditions. Unlike pure copper, which may promote some degree of lubrication but wears out more quickly, bronze is more durable and stable, which could contribute to increased material lifespan under tribological stress.

### 3.2. Tribological Behaviors of Basalt Fiber-Reinforced Hybrid Composites

The tribological performance data of basalt fiber-reinforced hybrid composites reveal the behavior of critical response variables—namely, the coefficient of friction, wear rate, and temperature—while also shedding light on the material’s degradation mechanisms in relation to the counterface material. The response variables of tribological tests of basalt fiber-reinforced hybrid composites, meaning wear specific rate, friction coefficient, and temperature are shown in Table 6.

**Figure 8:**
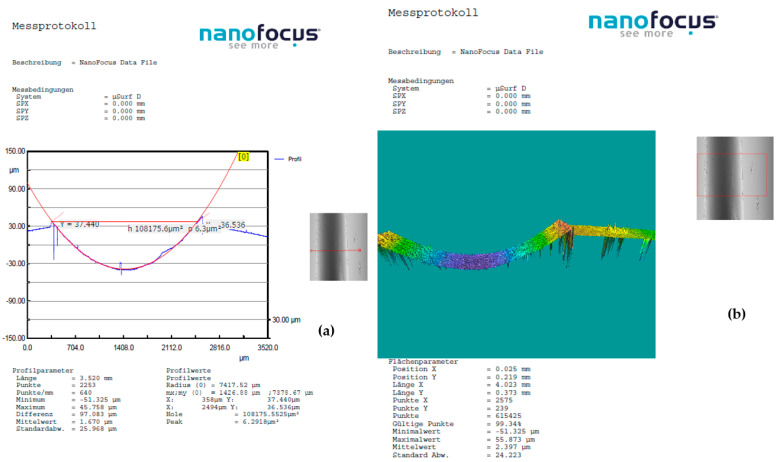
Dry friction test of BFRP + 5% CuSn10 sample, performed at 10 N load, 0.25 m/s sliding speed, and a duration of 120 min, in contact with a chromium–alloy steel ball: (**a**) Wear surface profile curve; (**b**) 3D worn surface morphology of the disk.

**Figure 9:**
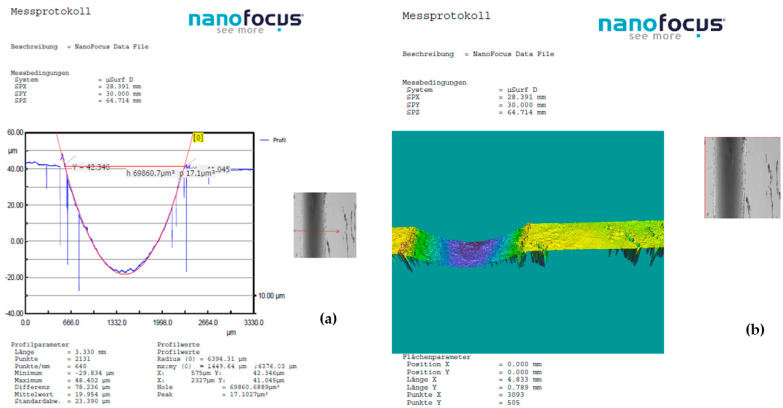
Dry friction test of BFRP + 10% CuSn10 sample, performed at 10 N load, 0.25 m/s sliding speed, and a duration of 120 min, in contact with a chromium–alloy steel ball: (**a**) Wear surface profile curve; (**b**) 3D worn surface morphology of the disk.

**Figure 10:**
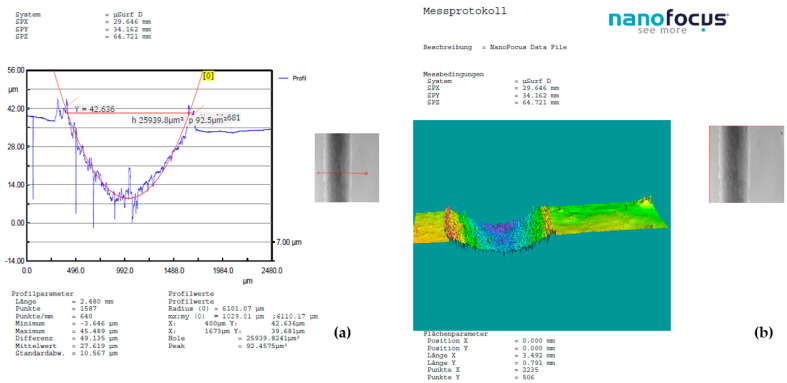
Dry friction test of BFRP + 15% CuSn10 sample, performed at 10 N load, 0.25 m/s sliding speed, and a duration of 120 min, in contact with a chromium–alloy steel ball: (**a**) Wear surface profile curve; (**b**) 3D worn surface morphology of the disk.

A detailed examination of surface modifications has yielded valuable insights into both the degradation mechanisms and the tribological performance of the tested material under dry friction conditions. To support these findings, Figure 8, Figure 9 and Figure 10 highlight representative 3D optical scans (performed using the ALICONA system—Optical 3D metrology & surface roughness measurement (Bruker Alicona, Graz, Austria)) of the worn surface morphology of composite disks reinforced with basalt fibers and varying concentrations of bronze powder (5%, 10%, and 15%). These visualizations highlight the influence of filler content on surface wear characteristics and contribute to a deeper understanding of material behavior during frictional contact.

The coefficients of variation (CVs) of the specific wear rate K were less than 13%, confirming the repeatability of the experiments. For the friction coefficient and temperature, coefficients of variation were found to be less than 2–3%, also confirming the repeatability of the experiments. Due to their very small values, these coefficients for COF and T were not highlighted in the experimental results tables in this work (Table 6).

**Figure 11:**
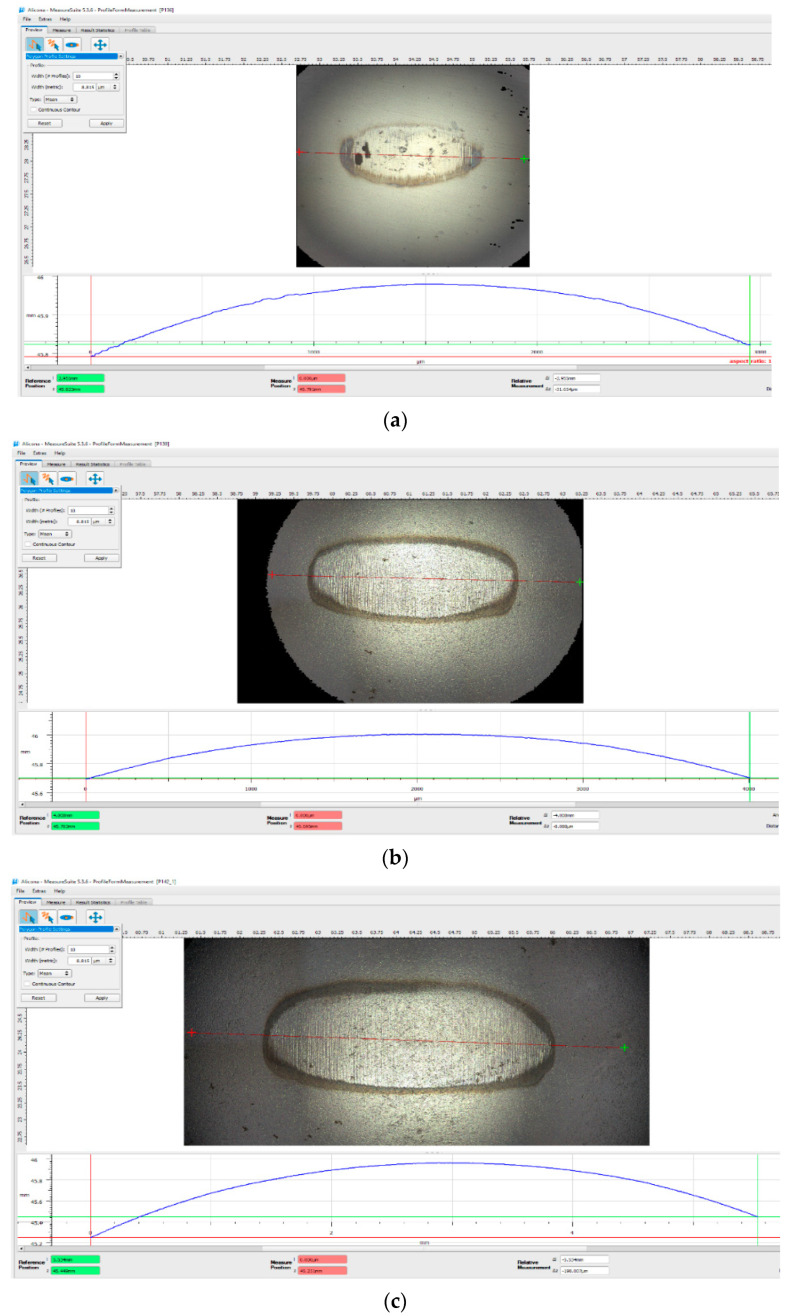
Wear marks on the chromium-alloyed steel ball after testing in contact with the BFRP + 15% CuSn10 disk, after a duration of 120 min, under different experimental conditions: (**a**) F = 10 N, v = 0.25 m/s; (**b**) F = 20 N, v = 0.25 m/s; (**c**) F = 30 N, v = 0.25 m/s.

The wear of the chromium-alloyed carbon steel 52100 ball was insignificant under all testing conditions, regardless of the concentration of bronze of composite material. Very little wear variation, depending on the the effects of applied force and sliding speed, were examined, as shown in Figure 11.

Table 7 presents the main statistical analysis results for the response variables: coefficient of friction, specific wear rate, and temperature. A control factor was deemed significant for a response variable if its *p*-value was below the threshold of 0.05.

The highest percentage contributions to the coefficient of friction (COF) were 52.43% for the weight fraction (wf) of CuSn10 powder, 22.21% for sliding speed (v), and 9.42% for the combined effect of applied force (F) and sliding speed (v), as shown in Table 7. For the specific wear rate, the CuSn10 powder weight fraction had the highest contribution at 45.58%, followed by the applied force at 33.71%, and the interaction between wf and v at 8.92%, as shown in Table 7.

**Figure 12:**
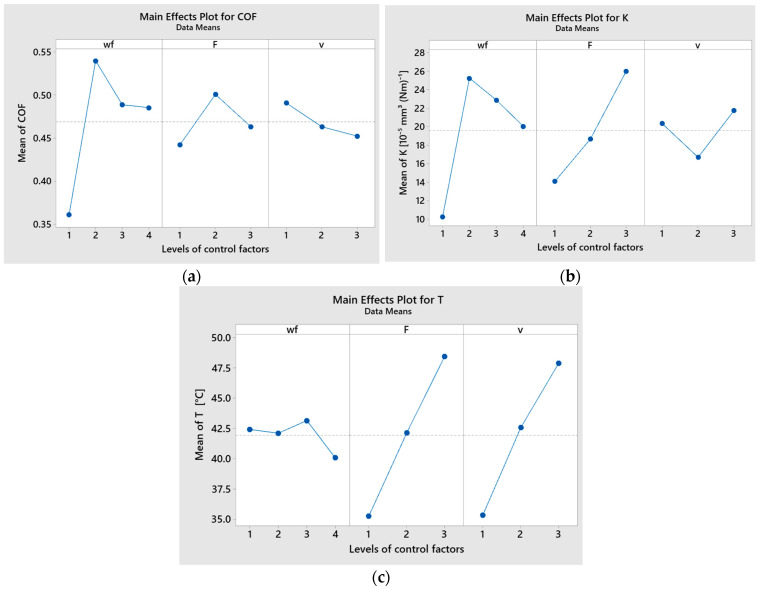
Main effects plot of CuSn10 powder, F, and v for (**a**) coefficient of friction; (**b**) specific wear rate; (**c**) temperature.

**Figure 13:**
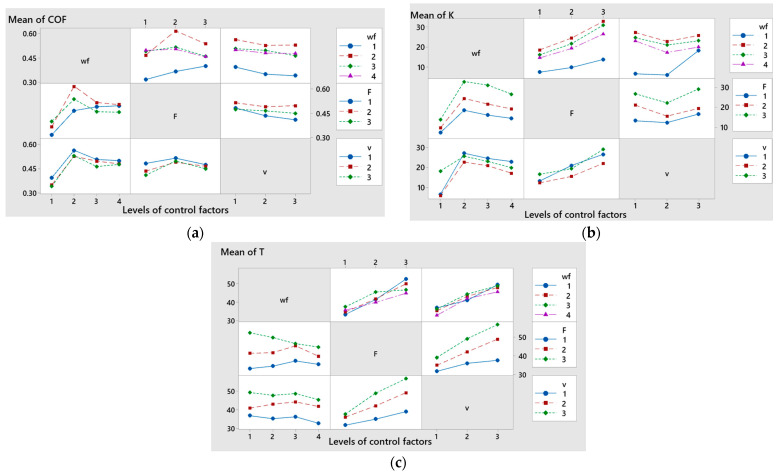
Interaction effects plot for (**a**) coefficient of friction; (**b**) specific wear rate; (**c**) temperature.

**Figure 14:**
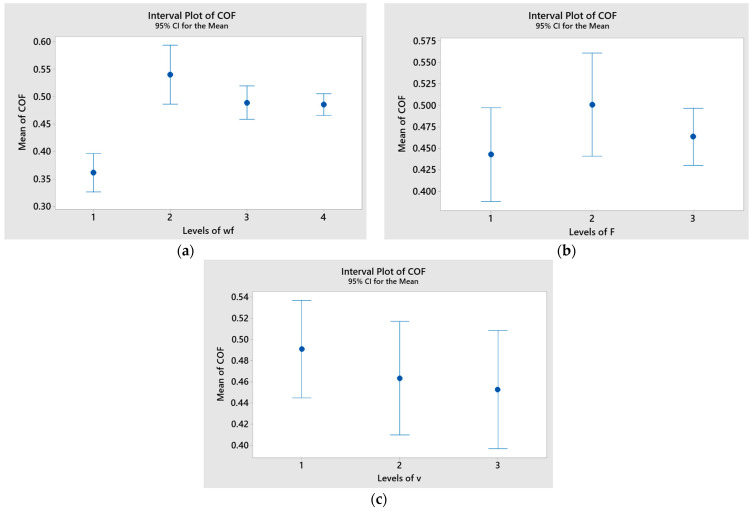
Interval plot of coefficient of friction (COF) factor of basalt fiber-reinforced hybrid composites with (**a**) wf; (**b**) F; (**c**) v. Individual standard deviations were used to calculate the interval plot. Bars are standard errors of the mean.

**Figure 15:**
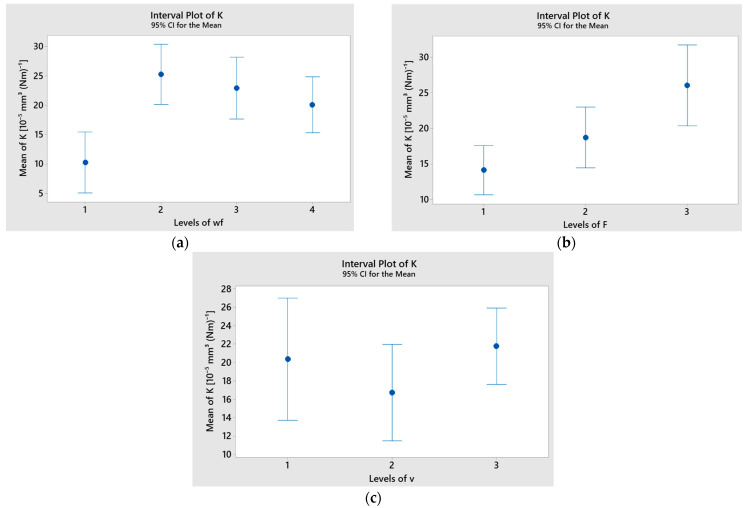
Interval plot of specific wear rate (K) factor of basalt fiber-reinforced hybrid composites with (**a**) wf; (**b**) F; (**c**) v. Individual standard deviations were used to calculate the interval plot. Bars are standard errors of the mean.

**Figure 16:**
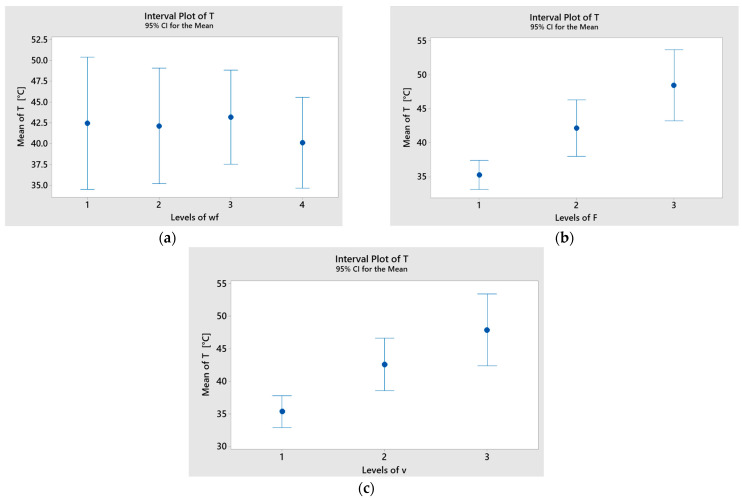
Interval plot of temperature (T) factor of basalt fiber-reinforced hybrid composites with (**a**) wf; (**b**) F; (**c**) v. Individual standard deviations were used to calculate the interval plot. Bars are standard errors of the mean.

The applied load and sliding speed were identified as the most significant factors influencing temperature, with contribution ratios of 43.88% and 39.93%, respectively. In contrast, the weight fraction of CuSn10 powder and the interaction effects between factors had comparatively lower impacts on temperature, according to the data in Table 6. Figure 12, Figure 13, Figure 14, Figure 15 and Figure 16 present the graphical outputs of the statistical analysis, including main effects, interaction, and interval plots for COF, specific wear rate (K), and temperature (T). The highest average values of the coefficient of friction (COF) were recorded for a CuSn10 powder weight fraction (wf) of 5%, applied force at level 2 (20 N), and sliding speed at level 1 (0.1 m/s), as shown in Figure 12a. A decreasing trend in COF was observed with increasing CuSn10 powder content. Additionally, COF increased with applied force up to 20 N and showed a decreasing trend with increasing sliding speed. The combination of wf = 5%, F = 20 N, and v = 0.1 m/s resulted in the highest mean COF values.

The main effects plot results for specific wear rate (K) indicate that the lowest average values were obtained at wf level 4 (15%), applied force level 1 (10 N), and sliding speed level 2 (0.25 m/s), as shown in Figure 12b. The specific wear rate decreased with an increase in the CuSn10 powder content but increased with rising applied load. Additionally, K showed a decreasing trend from sliding speed level 1 (0.1 m/s) to level 2 (0.25 m/s), followed by an increase at level 3 (0.36 m/s).

The main effects plot for temperature (T) indicated that the minimum mean values were achieved at 15% wf (level 4), 10 N applied force (level 1), and a sliding speed of 0.1 m/s (level 1), as shown in Figure 12c.

Figure 13a and Table 7 confirm that the coefficient of friction (COF) was significantly influenced by the interaction between applied force (F) and sliding speed (v). Similarly, the interaction between the weight fraction (wf) and sliding speed (v) had a notable effect on the specific wear rate, as shown in Figure 13b. Regarding temperature, significant influences were observed from the interaction between wf and F, as well as between F and v, as presented in Figure 13c.

The effect of each factor on the coefficient of friction (COF), along with standard error intervals, is visualized in Figure 14. A significant difference in the mean COF values for the weight fraction (wf) was observed, as the interval bar for level 1 (wf = 0%) did not overlap with those for levels 2–4 (Figure 14c). In contrast, the differences in mean COF values for applied force (F) and sliding speed (v) were likely not significant, as the standard error bars overlapped across all levels (Figure 14a,b).

Figure 15 displays the interval plots for each factor in relation to the specific wear rate (K). The minimum average values of K for the basalt fiber-reinforced hybrid composites were observed at wf = 15% (level 4), applied force F = 10 N (level 1), and sliding speed v = 0.25 m/s (level 2).

Figure 16 presents the interval plots for temperature across each factor, with the lowest mean values recorded at wf = 15%, F = 10 N, and v = 0.1 m/s. Significant differences between the mean temperature values for applied force (F) and sliding speed (v) were observed, as the corresponding interval bars did not overlap.

**Figure 17:**
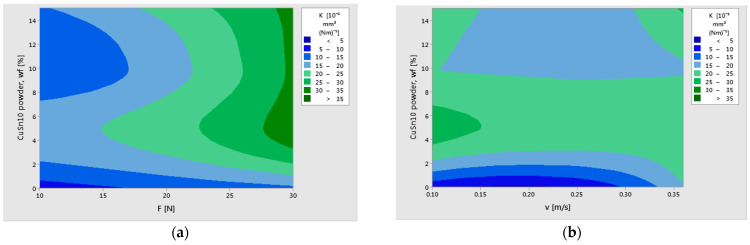
Contour plot prediction of specific wear rate (K) with (**a**) CuSn10 powder wf and F; (**b**) CuSn10 powder wf and v.

**Figure 18:**
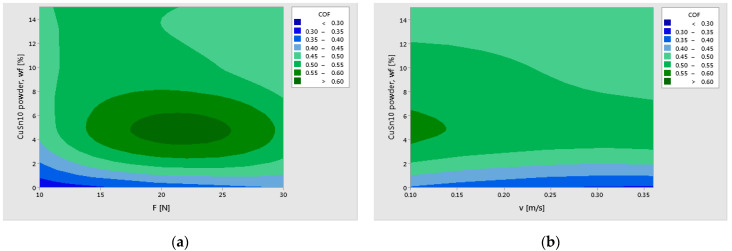
Contour plot prediction of coefficient of friction (COF) with (**a**) CuSn10 powder wf and F; (**b**) CuSn10 powder wf and v.

The normal probability plots of residuals for COF, K, and T validated the assumptions and adequacy of the Generalized Linear Models, as supported by reference [18]. The graphs from Figure 17 and Figure 18 have predicted the variation of K and COF, showing the relationship between the response variables (K and COF) and the control factors, CuSn10 powder (wf%), applied force (F), and sliding speed (v).

Figure 17 illustrates the predicted behavior of the specific wear rate (K) as a function of CuSn10 reinforcement content and the two key operational parameters: applied load (F) and sliding speed (v). In subplot (a), it is evident that the wear rate decreases significantly at lower loads (10 N) and higher bronze powder content (15 wt%). However, for intermediate loads (20 N), the wear rate tends to increase again for mid-level filler contents, suggesting a potential instability in film formation or local thermal effects. This nonlinear interaction between reinforcement level and contact pressure reveals the complex wear mechanisms at play.

In subplot (b), the influence of CuSn10 content and sliding speed on wear rate is highlighted. The wear rate appears minimized at medium speeds (around 0.25 m/s), particularly for 10–15 wt% CuSn10, where a stable transfer layer is likely formed. At higher speeds and lower filler contents, wear rate increases again, possibly due to surface overheating and insufficient lubrication. These contour plots provide a valuable prediction tool and could benefit further from the inclusion of optimized regions that are marked explicitly and possibly overlap with experimental data points for visual validation.

Figure 18 presents the predicted variation of the coefficient of friction (COF) with respect to the CuSn10 filler content, applied load, and sliding speed. In subplot (a), the COF peaks at 5 wt% CuSn10 under 20 N load, which aligns with experimental observations. As the CuSn10 content increases to 10–15 wt%, the COF decreases substantially, indicating the formation of a more stable oxide layer that acts as a solid lubricant. Interestingly, at higher loads (30 N), the friction reduction becomes less pronounced, likely due to the breakdown of this lubricating layer under high thermal and mechanical stress.

In subplot (b), the relationship between COF and sliding speed is explored. A nearly linear decrease in friction is observed as the speed increases, particularly for composites with higher CuSn10 content. This trend confirms the synergistic effect of bronze particles and dynamic frictional heating, which promotes the formation of a protective tribolayer. Enhancing the graphical contrast and indicating the optimum tribological regions would make these visualizations even more impactful.

### 3.3. Morphology Analysis

The SEM images and EDS spectra from the analysis of the BFRP_CuSn10 samples provide essential information about the microstructural and chemical changes that occurred following the tribological tests. These results are crucial for understanding the wear mechanisms and for correlating the tribological performance of the composite materials with their microscopic structure.

**Figure 19:**
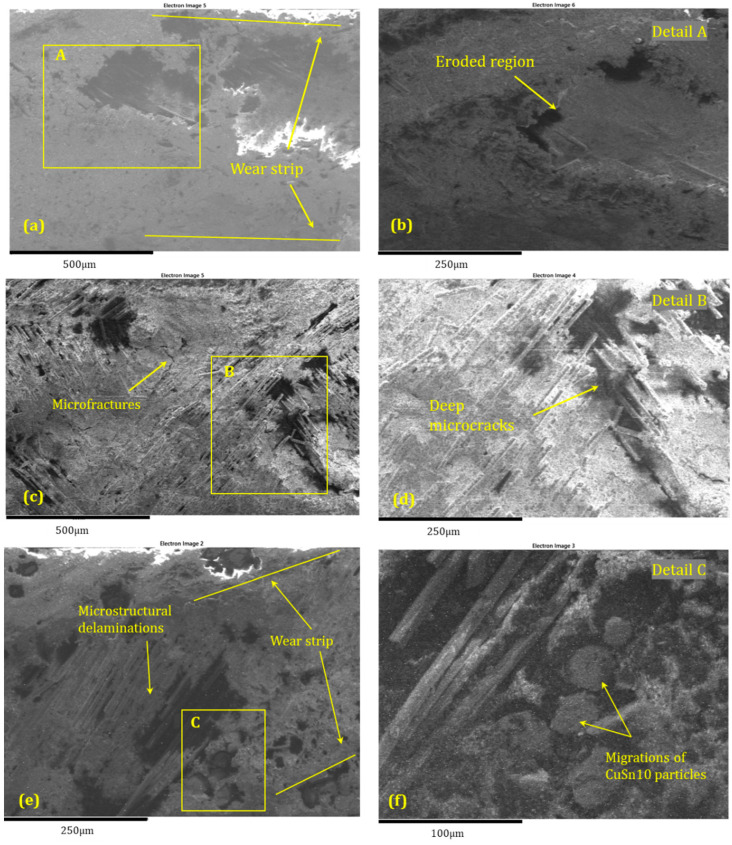
SEM micrographs of wear tracks on BFRP composites with CuSn10 content of (**a**,**b**) 5%, (**c**,**d**) 10%, and (**e**,**f**) 15%, following 120 min of dry sliding against a 52100 steel ball at 10 N load and 0.25 m/s sliding speed.

The SEM analysis of the worn and unworn surfaces of the BFRP_CuSn10 samples at the three concentrations shows significant differences between the initial state and the state after tribological testing (Figure 19a–f). Although a side-by-side comparative arrangement could enhance visual synthesis, we chose to present each SEM image separately to maintain clarity in observing distinct surface features for each CuSn10 content. This approach facilitates focused analysis of individual wear mechanisms and avoids visual congestion, especially given the detailed morphological differences observed. The unworn surface presents a homogeneous morphology without significant imperfections, indicating a uniform distribution of bronze particles in the composite matrix. On the other hand, the worn surface is marked by the presence of parallel scratches and grooves oriented in the sliding direction, which is a clear sign of an abrasive wear mechanism. Microcracks and areas of local delamination can also be observed, suggesting progressive degradation of the surface layer from friction. In addition, the presence of adherent particles on the surface indicates a possible material transfer process between the contact surfaces.

**Figure 20:**
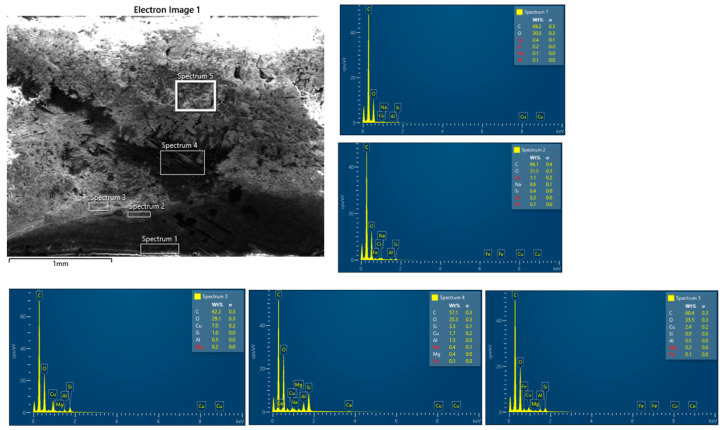
EDS analysis of the BFRP_CuSn10_5% sample after 120 min of operation at 10 N load and 0.25 m/s sliding speed.

**Figure 21:**
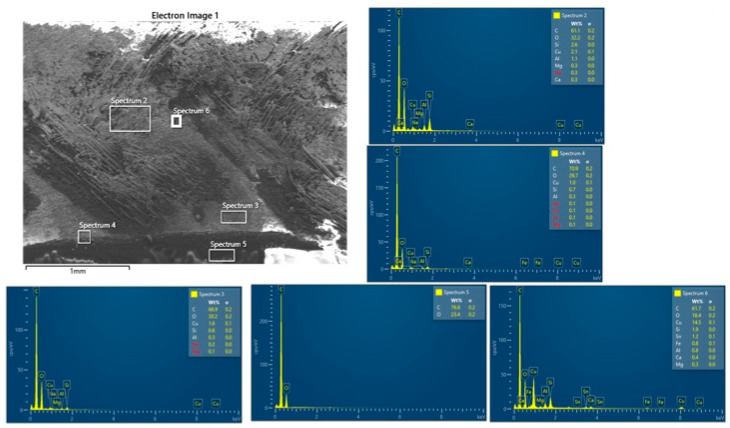
EDS analysis of the BFRP_CuSn10_10% sample after 120 min of operation at 10 N load and 0.25 m/s sliding speed.

**Figure 22:**
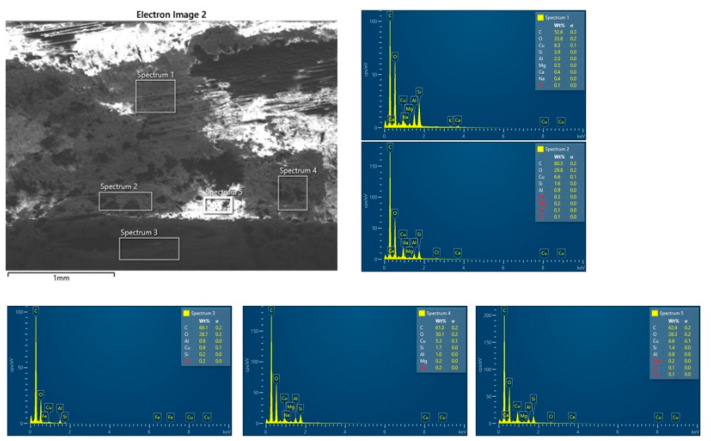
EDS analysis of the BFRP_CuSn10_15% sample after 120 min of operation at 10 N load and 0.25 m/s sliding speed.

The EDS spectroscopy performed on the BFRP_CuSn10 samples at different concentrations (5%, 10%, and 15%), presented in Figure 20, Figure 21 and Figure 22, and confirms the presence of Cu and Sn elements, demonstrating the distribution of bronze in the composite structure. Although EDS mapping was not performed, the point and area spectra were collected directly from worn regions where tribolayer formation was observed, providing localized evidence of Cu, Sn, and O presence associated with oxide-based tribofilms. An important aspect observed in the chemical analysis is the presence of a significant oxygen content, which suggests the formation of metal oxides during the friction process. These oxides may aid in forming a protective layer on the composite surface, which positively influences the material’s tribological performance. Traces of Fe and Cr were also identified, indicating a possible material transfer from the 52100 steel ball used in the testing.

By comparing the samples with different bronze concentrations, it is observed that the samples with 10% and 15% CuSn10 exhibit a more stable protective layer, which reduces severe wear and the coefficient of friction. In the case of these samples, the SEM analysis shows a less affected surface, with fewer deep scratches and a lower degree of damage. This observation is supported by the EDS analysis, which highlights an increase in the amount of metal oxides (CuO, SnO_2_), suggesting that the oxidation process plays a key role in reducing wear.

Therefore, it can be concluded that the dominant wear mechanism in samples with a low bronze content is abrasive wear, characterized by deep scratches and severe surface degradation. On the other hand, samples with a higher CuSn10 content benefit from a protective layer formed from bronze and metal oxides, which leads to reduced wear and improved tribological performance.

In addition, the interaction between the basalt particles and the bronze layer contributes to filling the microcavities on the contact surface, thus reducing direct material transfer and premature damage. These results demonstrate the importance of optimizing the composition of the composite material to achieve superior tribological performance. In particular, adding an appropriate percentage of bronze can contribute to the formation of an effective protective film, reducing wear and improving the stability of the friction regime.

Thus, the combined SEM and EDS analysis provides a detailed perspective on the wear behavior of BFRP_CuSn10 composites and allows the identification of effective strategies for improving the durability of these materials in tribological applications.

## 4. Conclusions

This study investigated the effect of CuSn10 bronze powder (5%, 10%, and 15% wt) on the mechanical and tribological performance of basalt fiber-reinforced polymer (BFRP) composites. The results clearly show that the addition of CuSn10 significantly enhances both tensile properties and wear resistance, especially at 10–15 wt% concentrations.

The highest tensile strength (440.6 MPa) was recorded for the composite with 10% CuSn10, indicating improved load transfer and interfacial bonding due to the metallic filler. Meanwhile, the best tribological performance—characterized by the lowest coefficient of friction and specific wear rate—was achieved with 15% CuSn10 under a 10 N load and 0.25 m/s sliding speed. This improvement was attributed to the formation of a stable oxide-based tribolayer (CuO and SnO_2_), as confirmed by SEM and EDS analyses, which reduced surface degradation and stabilized friction.

Statistical analysis using GLM models showed that the CuSn10 content had the highest influence on both the friction coefficient (52.43%) and wear rate (45.58%), followed by load and sliding speed. These results confirm the critical role of filler concentration in optimizing tribological behavior.

In summary, BFRP composites reinforced with CuSn10, especially at 15 wt%, offer a well-balanced combination of strength, durability, and frictional stability. These hybrid materials are well suited for structural and tribological applications in sectors such as automotive, aerospace, and mechanical systems where dry sliding and wear resistance are essential.

While 10 wt% CuSn10 resulted in the highest tensile strength and mechanical homogeneity, 15 wt% CuSn10 exhibited the most favorable tribological performance in terms of wear resistance and friction stability. These findings suggest a trade-off between mechanical strength and tribological efficiency, and the optimal composition should therefore be selected based on the specific performance priorities of the intended application.

## Figures and Tables

**Table 1:** Designation and compositional details of laminated composite specimens.

Laminated Composite of Basalt Fibers, Epoxy, and CuSn10 Bronze Powder	Basalt Fiber (wf%)	Epoxy Resin (wf%)	Bronze Powder (wf%)
BFRP + 0% CuSn10	50	50	0
BFRP + 5% CuSn10	50	45	5
BFRP + 10% CuSn10	50	40	10
BFRP + 15% CuSn10	50	35	15

**Table 2:** Experimental parameters used in tribological tests.

Parameters	Test Conditions
Load (Force)	10, 20, 30 N
Sliding Speed	0.1, 0.25, 0.36 m/s
Relative Humidity	45 (±2)%
Initial Temperature	22 (±1) °C
Test Duration	120 min
Conditions	Dry friction
Disk/Ball Materials	Basalt fiber-reinforced polymer composites with CuSn10 powder additives tested using alloyed chromium steel balls (AISI 52100).
Average Surface Roughness (Ra disk/ball)	* 0.60–0.66 for basalt fiber disks/0.02 µm

Note: * denotes different surface roughness values according to the type of composite material employed.

**Table 3:** Control factors and their levels for response analysis.

Response Variables	CuSn10 Powder, wf		Applied Load, F		Sliding Speed, v	
	Symbol	Value [%]	Symbol	Value [N]	Symbol	Value [m/s]
Coefficient of friction (COF) Specific wear rate (K) Temperature (T)	1	0	1	10	1	0.10
	2	5	2	20	2	0.25
	3	10	3	30	3	0.36
	4	15	-	-	-	-

**Table 4:** Tensile properties of basalt fiber-reinforced composites with bronze powder.

Sample BFRP 50% + %CuSn10	Tensile Strength (MPa) /SD (MPa)/CV (%)	Tensile Strain at Maximum Load (%) /SD (%)/CV (%)	Elastic Modulus E (GPa)/SD (MPa)/CV(%)
0% CuSn10	329.2/(29.3)/8.89	1.84/(0.05)/2.7	20,840/(2240)/10.73
5% CuSn10	414.4/(37.8)/9.13	2.5/(0.39)/15.41	18,960/(941)/4.96
10% CuSn10	440.6/(18.6)/4.22	2.62/(0.15)/5.68	18,400/(377)/2.04
15% CuSn10	399.4/(18.1)/4.53	2.47/(0.14)/5.5	17,740/(337)/1.9

Note: SD—Standard deviation and CV—Coefficient of variation of the results.

**Table 5:** Flexure mechanical properties of basalt fiber-reinforced composites with bronze powder.

Sample BFRP 50% + %CuSn10	Rezistența la Încovoiere (MPa)/SD (MPa)/CV(%)	Deformația la Încovoiere (%)/SD (%)/CV (%)	Modulul de Elasticitate E (GPa)/SD (MPa)/CV(%)
0% CuSn10	284.31/(26.81)/9.42	2.22/(0.16)/7.20	20,200.2/(2410)/11.89
5% CuSn10	295/(18.6)/6.30	2.31/(0.4)/1.73	18,700/(1460)/7.82
10% CuSn10	280.41/(11.24)/4	1.41/(0.56)/3.97	21.740/(2100)/9.69
15% CuSn10	273.23/(15.36)/5.62	2.3/(0.26)/1.13	20,500/(747)/3.64

Note: SD—Standard deviation and CV—Coefficient of variation of the results.

**Table 6:** Experimental tribological properties for the BFRP_CuSn10/chromium-alloyed carbon steel (52100) coupling.

Experimental Parameters			Mean of Measured Parameters			
Exp.no.	Load F (N)	Sliding Speed v [ms^−1^]	CuSn10 Powder Content, wf [%]	Wear Specifical Rate K [10^−5^ mm^3^ × (Nm)^−1^]/CV [%]	Friction Coefficient COF *	Average Temperature T * [°C]
1	10	0.1	0	3.247/8.85	0.36	30.9
2	10	0.1	5	18.3/1.52	0.52	32.4
3	10	0.1	10	10.81/10.17	0.53	34.6
4	10	0.1	15	15.16/3.57	0.52	29.5
5	10	0.25	0	2.695/4.89	0.31	32.4
6	10	0.25	5	17.578/8.03	0.43	35.2
7	10	0.25	10	10.182/10.17	0.52	38.2
8	10	0.25	15	13.99/7.84	0.48	38.9
9	10	0.36	0	16.022/5.23	0.28	36.9
10	10	0.36	5	19.6126/8.39	0.45	36.3
11	10	0.36	10	10.6861/11.1	0.42	39.5
12	10	0.36	15	14.2792/7.84	0.49	38.4
13	20	0.1	0	7.651/8.76	0.4	27
14	20	0.1	5	28.0098/8.52	0.63	33.4
15	20	0.1	10	17.1008/8.48	0.52	37
16	20	0.1	15	23.3253/11.06	0.51	33
17	20	0.25	0	5.8449/9.23	0.34	38
18	20	0.25	5	20.3379/6.48	0.6	43.3
19	20	0.25	10	9.3767/5.6	0.51	46.5
20	20	0.25	15	17.2705/8.53	0.51	41.1
21	20	0.36	0	9.09/7.56	0.36	49.3
22	20	0.36	5	20.6124/3.6	0.62	48.7
23	20	0.36	10	16.8999/10.66	0.52	52.8
24	20	0.36	15	24.845/4.98	0.49	45.5
25	30	0.1	0	8.693/9.92	0.42	42.9
26	30	0.1	5	35.3431/13.33	0.54	40.2
27	30	0.1	10	32.2748/10.75	0.47	37.4
28	30	0.1	15	35.579/6.49	0.47	33.1
29	30	0.25	0	9.2219/6.59	0.4	58.60
30	30	0.25	5	30.43025/11.89	0.55	50.8
31	30	0.25	10	28.7675/6.63	0.46	45.3
32	30	0.25	15	20.276/9.7	0.45	45.6
33	30	0.36	0	23.29/9.68	0.38	65
34	30	0.36	5	32.71388/11.75	0.52	58.7
35	30	0.36	10	31.9356/9.71	0.45	54.1
36	30	0.36	15	38.6308/5.15	0.45	52.7

Note: CV—Coefficient of variation of the results; * average of the last 60 min.

**Table 7:** The statistical results for coefficient of friction, specific wear rate, and temperature.

		COF			K			T	
Source	F-Value	*p*-Value	PC [%]	F-Value	*p*-Value	PC [%]	F-Value	*p*-Value	PC [%]
wf	38.80	<0.001	52.43	157.09	<0.001	45.58	6.57	0.007	1.95
F	0.51	0.612	0.46	174.27	<0.001	33.71	221.22	<0.001	43.88
v	23.55	<0.001	21.21	32.66	<0.001	6.32	201.3	<0.001	39.93
wf*F	1.85	0.173	4.99	4.62	0.012	2.68	9.58	0.001	5.7
wf*v	2.25	0.109	6.08	15.37	<0.001	8.92	1.89	0.163	1.13
F*v	5.23	0.011	9.42	4.22	0.023	1.63	15.67	<0.001	6.22
Error	-	-	5.4	-	-	1.16	-	-	1.19
Total	-	-	100	-	-	100	-	-	100

## Data Availability

Original contributions presented in this study are included in this article; further inquiries can be directed to the corresponding authors.
